# Preventive effect of dietary astaxanthin on UVA-induced skin photoaging in hairless mice

**DOI:** 10.1371/journal.pone.0171178

**Published:** 2017-02-07

**Authors:** Toshiyuki Komatsu, Suguru Sasaki, Yuki Manabe, Takashi Hirata, Tatsuya Sugawara

**Affiliations:** Graduate School of Agriculture, Kyoto University, Kyoto, Japan; University of Alabama at Birmingham, UNITED STATES

## Abstract

Astaxanthin, a carotenoid found mainly in seafood, has potential clinical applications due to its antioxidant activity. In this study, we evaluated the effect of dietary astaxanthin derived from *Haematococcus pluvialis* on skin photoaging in UVA-irradiated hairless mice by assessing various parameters of photoaging. After chronic ultraviolet A (UVA) exposure, a significant increase in transepidermal water loss (TEWL) and wrinkle formation in the dorsal skin caused by UVA was observed, and dietary astaxanthin significantly suppressed these photoaging features. We found that the mRNA expression of lympho-epithelial Kazal-type-related inhibitor, steroid sulfatase, and aquaporin 3 in the epidermis was significantly increased by UVA irradiation for 70 days, and dietary astaxanthin significantly suppressed these increases in mRNA expression to be comparable to control levels. In the dermis, the mRNA expression of matrix metalloprotease 13 was increased by UVA irradiation and significantly suppressed by dietary astaxanthin. In addition, HPLC-PDA analysis confirmed that dietary astaxanthin reached not only the dermis but also the epidermis. Our results indicate that dietary astaxanthin accumulates in the skin and appears to prevent the effects of UVA irradiation on filaggrin metabolism and desquamation in the epidermis and the extracellular matrix in the dermis.

## Introduction

The skin functions as the outermost barrier of the body and is in direct contact with the environment, which causes physical damage. Skin can regulate the local and global homeostasis by sensing the environment [1, 2]. However, chronic exposure to ultraviolet (UV) radiation from the sun contributes to skin photoaging, which is clinically characterized by dryness, pigmentation, laxity, and deep wrinkling [3, 4]. UV radiation comprises wavelengths from 200 to 400 nm, and is further divided into three sections: UVA (320–400 nm), UVB (280–320 nm), and UVC (200–280 nm). Although UVC is filtered out by atmospheric ozone for the most part, both UVA and UVB radiation can reach the Earth’s surface and cause biological consequence to the skin [5, 6]. UVB radiation critically damages cellular macromolecules and induces the formation of reactive oxygen species. Exposure to UVB radiation is the primary cause of skin cancer in humans and animals [7]. Despite being weakly carcinogenic as compared to UVB, UVA radiation contributes up to 95% of the total UV exposure and plays a substantial role in photoaging of the human skin [8]. UVA is able to penetrate the dermis, resulting in damage to dermal collagen and elastin, while UVB mainly affects the epidermis and causes DNA damage [9]. Therefore, dietary supplementation with molecules that can effectively accumulate in the dermis could protect the skin from UVA-induced damage such as photoaging.

Astaxanthin, 3,3’-dihydroxy-β-carotene-4,4’-dione, is a carotenoid found mainly in seafood such as salmon, trout, lobster, shrimp, and fish eggs [10, 11]. It is well known that astaxanthin has potential clinical applications due to its antioxidant activity, which is higher than that of α-tocopherol and other carotenoids including β-carotene [12, 13]. Furthermore, unlike other antioxidants, astaxanthin exerts antioxidative effects without being pro-oxidative [14]. Astaxanthin possesses many highly potent pharmacological effects, including anti-tumor, anti-cancer, anti-diabetic, anti-atherosclerotic, and anti-inflammatory activities [15–17]. In addition, it was also reported that treatment with astaxanthin prevents UV-induced photokeratitis in mice by decreasing the oxidative stress in the irradiated eyes [18] and that atopic dermatitis was improved by oral administration of astaxanthin via the regulation of the inflammatory effects and the expression of inflammatory cytokines in a murine model [19]. In cultured human skin fibroblasts, astaxanthin prevented UVA-induced DNA damage [20]. Therefore, dietary astaxanthin is expected to contribute the prevention of skin photoaging, if it can accumulate in the dermis. However, there is no information about the effect of dietary astaxanthin on skin photoaging *in vivo*.

In this study, we evaluated the effect of dietary astaxanthin derived from *Haematococcus pluvialis* on skin photoaging in UVA-irradiated hairless mice by determining various parameters of photoaging. *H*. *pluvialis* is a green microalga, which accumulates high concentrations of astaxanthin under several stress conditions, and is a primary source of astaxanthin used in the food industry and aquaculture [21, 22]. Our results indicate that dietary astaxanthin effectively prevents skin photoaging caused by UVA exposure in mice.

## Materials and methods

### Materials

Astaxanthin monoester was purified from a commercially available oil extracted from *H*. *pluvialis* (ASTOTS-S, FUJIFUILM Co., Tokyo, Japan) by silica gel column [23]. Briefly, the oil was dissolved in hexane/chloroform (5/1, v/v) and loaded on a chromatographic column (50×300 mm) packed with 160 g of silica gel in hexane. The column was flashed with hexane and acetone/hexane (1/10, v/v). The astaxanthin monoester fraction eluted with acetone/hexane (1/5) was collected and then applied to silica TLC developed in chloroform/methanol/water (64/16/2, v/v/v) for checking the purity.

### Animals

Female hairless Hos:HR-1 mice (6 weeks old) were purchased from Hoshino Laboratory Animals (Ibaragi, Japan). They were housed at 24 ± 1°C with a 12-h light:dark cycle. They were fed with free access to standard chow (Oriental Yeast, Tokyo, Japan) and water during the experiments. The experimental protocol of this study was approved by the Kyoto University animal committee. After acclimatization, mice were divided into four groups (n = 5). Mice in experimental groups were fed a diet of AIN-93G with 0.01% or 0.1% astaxanthin monoester purified from *H*. *pluvialis* (0.01%Ax and 0.1%Ax groups) (Table 1). Mice in normal and control groups were fed AIN-93G alone.

**Table 1:** Composition of experimental diets.

Ingredient	Normal	Control	0.01%Ax	0.1%Ax
	%			
Cornstarch	39.7486	39.7486	39.7486	39.7486
Casein	20.0	20.0	20.0	20.0
Dextrinized corn starch	13.2	13.2	13.2	13.2
Sucrose	10.0	10.0	10.0	10.0
Soybean oil	7.0	7.0	6.99	6.9
Cellulose powder	5.0	5.0	5.0	5.0
AIN-93G mineral	3.5	3.5	3.5	3.5
AIN-93G vitamin	1.0	1.0	1.0	1.0
ʟ-Cystine	0.3	0.3	0.3	0.3
Choline bitartrate	0.25	0.25	0.25	0.25
Buthyl hydroxy toluene	0.0014	0.0014	0.0014	0.0014
Astaxanthin monoester	0	0	0.0144	0.1436
(Astaxanthin equivalent)			(0.01)	(0.1)
Total	100	100	100	100

### Animal experiment

The UV source was a bank of two UV lamps BLB (15 W, maximum emission intensity at 365 nm, UVP, California, USA). The distance from the lamps to the mice was approximately 18 cm, and fans circulated the air. Irradiation was carried out in an air-conditioned room, and the temperature in the cage was maintained at 23–26°C. Mice in the experimental groups were exposed to a dose of 20 J/cm^2^ five times weekly for 70 days [24]. UV strength was measured at 365 nm with a UV radiometer VLX-3W (Cosmo Bio, Tokyo, Japan). No irradiation was performed in the normal group. At the end of the 70 days, the mice were sacrificed under isoflurane anesthesia. Blood and dorsal skin specimens were collected immediately. The pieces of dorsal skin were fixed in 10% neutral buffered formalin solution for morphological analysis and the dorsal skin sections were stained with hematoxylin and eosin. For analysis of mRNA expression, the skin specimens were stored in RNAlater (Qiagen, Valencia, CA) at -80°C until use.

### Evaluation of wrinkle formation in the dorsal skin

Dorsal skin collected at 28, 56, and 70 days was replicated by using a silicone product (Asahi Biomed, Yokohama, Japan) under isoflurane anesthesia [25]. Images of the skin replica were analyzed using skin wrinkle analysis software (Asahi Biomed). The parameters to assess skin wrinkles were total groove volume ratio, wrinkle area ratio, wrinkle volume ratio, and the number of wrinkles.

### HPLC analysis of astaxanthin concentration in the skin and plasma

To separate skin epidermis from dermis, mouse dorsal skin collected using disposable biopsy punches (Kai Industries Co., Ltd., Gifu, Japan) was reacted with 2.5 U/mL Dispase^®^ II (neutral protease, grade II, Roche, Indianapolis, IN) in Hanks’balanced salt solution+ (HBSS(+), Nacalai Tesque, Inc., Kyoto, Japan) overnight at 4°C [26]. After incubation, the epidermis was separated from the dermis at the basement membrane. Total lipids were extracted from each epidermis and dermis sample, as well as from plasma samples, by using chloroform and methanol [27]. After evaporation of the collected chloroform phase, the residue was dissolved in an aliquot of methanol and then subjected to HPLC analysis for the quantification of astaxanthin [23].

### Quantification of NMFs by HPLC

To determine the content of NMFs in the epidermis, we analyzed PCA and UCA by HPLC. The mouse epidermis (0.5 cm^2^), prepared as described above, was homogenized with 100 μL of saline and then added to 300 μL of ethanol. After mixing vigorously for 1 min and centrifuging (1,700×g) for 15 min at 4°C, the supernatant was collected and then evaporated by nitrogen. The sample was dissolved in distilled water, and the aliquot was applied to HPLC equipped with a photodiode array detector with a COSMOSIL 5C18AR column (3.0×150 mm, 5 μm, Nacalai Tesque). The mobile phase was water adjusted to pH 2.6 using phosphoric acid with a flow rate of 0.4 mL/min. PCA and UCA were detected at 210 and 270 nm, respectively, and quantified at their peak area by using a standard curve with authentic standards.

### RNA preparation and real-time qRT-PCR

Skin samples in RNAlater were washed with HBSS (+) and then separated into epidermis and dermis as described above. Total RNA was extracted from the epidermis and dermis using Sepasol reagent (Nacalai Tesque) according to the manufacturer’s instructions and treated with DNase (Wako Pure Chemical Industries). cDNAs were synthesized using SuperScript RNase II reverse transcriptase (Invitrogen, Carlsbad, CA) with random hexamers. For RT-PCR, cDNA was diluted and mixed with iQ SYBR Green Supermix (Bio-Rad Laboratories) containing 1 mmol/L PCR primer (primer sequences are shown in S1 Table). Real-time qRT-PCR was performed by using a DNA Engine Option system (Bio-Rad Laboratories). The thermal cycling conditions were 15 min at 95°C for 1 cycle, followed by amplification for 43 cycles with melting for 15 s at 95°C and annealing and extension for 30 s at 60°C. The expression level of each gene was normalized by using GAPDH as an internal control.

### Statistical analysis

Data are presented as the mean ± SD. Statistical analyses were carried out by one-way ANOVA followed by Scheffe test. Differences were considered significant for values of P < 0.05.

## Results

### Effect of dietary astaxanthin on skin properties and other tissues

Daily food intake and body weight gain were not significantly different among the four groups during the experimental period (Table 2). After the treatment for 70 days, tissue weights excluding mesenteric white adipose tissue were not significantly different among the groups. The weight of mesenteric adipose tissue in 0.01%Ax group was significantly lower than that in control group. There was no difference in plasma parameters among groups (Table 3).

**Table 2:** Total food intake, body weight gain and tissue weight in mice.

	Normal	Control	0.01%Ax	0.1%Ax
Total food intake (g)	256 ± 13	241 ± 13	246 ± 10	255 ± 16
Body weight gain (g)	5.2 ± 1.8	5.0 ± 1.8	4.2 ± 1.1	3.9 ± 1.0
Tissue weight (g)				
Liver	1.13 ± 0.11	1.38 ± 0.29	1.08 ± 0.08	1.27 ± 0.19
Spleen	0.070 ± 0.010	0.085 ± 0.024	0.070 ± 0.006	0.087 ± 0.039
Heart	0.120 ± 0.017	0.122 ± 0.008	0.123 ± 0.015	0.127 ± 0.018
Kidney	0.352 ± 0.045	0.335 ± 0.037	0.348 ± 0.040	0.330 ± 0.025
Adipose tissue				
Mesenteric	0.454 ± 0.063^a,b^	0.503 ± 0.046^a^	0.277 ± 0.103^b^	0.300 ± 0.061^a,b^
Perirenal	0.240 ± 0.137	0.200 ± 0.051	0.208 ± 0.130	0.195 ± 0.077

Values are mean ± SD, n = 5. Values in rows with different letters significantly different (P < 0.05).

**Table 3:** Plasma parameters in mice.

	Normal	Control	0.01%Ax	0.1%Ax
Glucose (mg/dL)	196±60	225±91	214±48	239±76
Triacylglycerol (mg/dL)	50±9	48±19	55±16	57±15
Aspartate aminotransferase (IU/L)	46±18	53±27	36±5	53±25
Alanine aminotransferase (IU/L)	2.6±1.6	2.0±1.1	1.8±0.6	1.8±0.4

Values are mean ± SD, n = 5.

**Fig 1:**
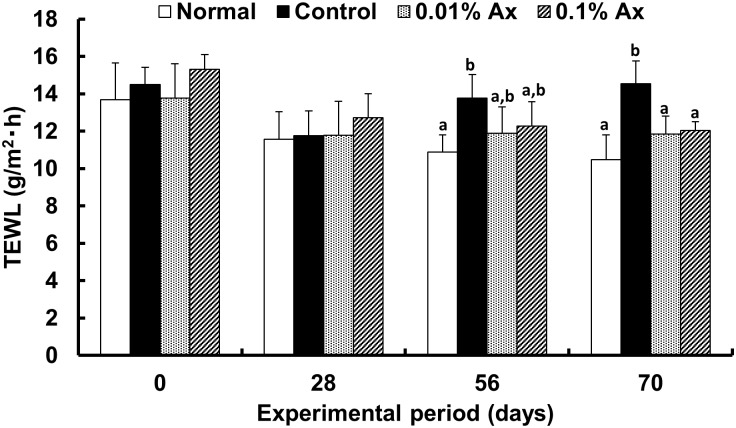
Effect of dietary astaxanthin on TEWL levels in the hairless mice. Data represent the means ± SD (n = 5). Bars with matching letters at each time point are significantly different from each other at P < 0.05.

To evaluate the epidermal permeability barrier function, transepidermal water loss (TEWL) was periodically monitored during the UVA-irradiation period (Fig 1). After chronic UVA exposure, a significant increase in TEWL by UVA was observed, and which demonstrated a trend toward suppression by dietary astaxanthin at 56 days. At 70 days, TEWL in both the 0.01% and 0.1% astaxanthin-supplemented groups was significantly lower than that in the control UVA-irradiated group, but the effect was not dose dependent.

**Fig 2:**
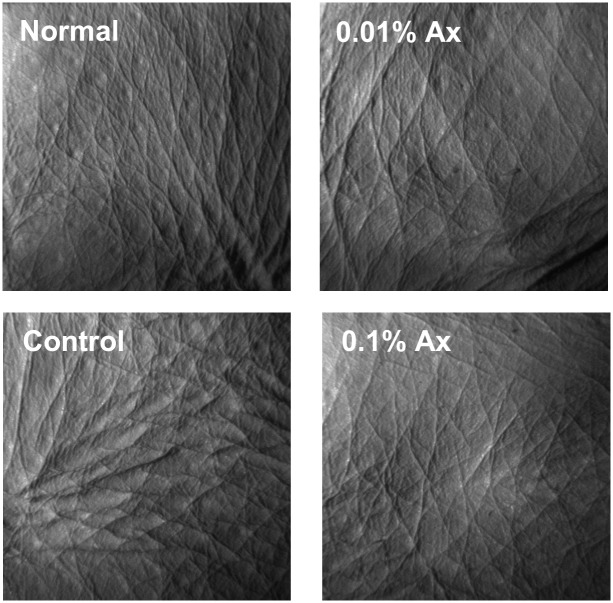
Photographs of replicas taken from the dorsal skin of the hairless mice.

**Fig 3:**
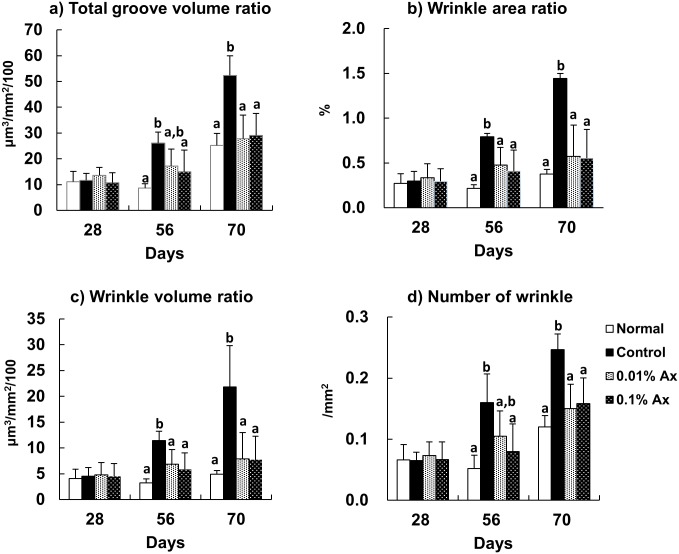
Effect of dietary astaxanthin on UVA-induced wrinkle formation in the hairless mice. Data represent the means ± SD (n = 5). Bars with matching letters at each time point are significantly different from each other at P < 0.05.

To compare the level of wrinkle formation, skin replicas were collected at 28, 56, and 70 days. Fig 2 shows enlarged images of skin replicas from representative mice in each group at 70 days. In the control group, deep coarse wrinkles were formed. To quantify the degree of wrinkle formation, skin replicas were analyzed using a 3-D image analysis system (Fig 3). Total groove volume ratio, wrinkle area ratio, wrinkle volume ratio, and the number of wrinkles were significantly increased in the control group compared with the normal group, and dietary astaxanthin significantly suppressed or trended toward suppressing the increases of these parameters by UVA irradiation at 56 days. These parameters were significantly lower in all astaxanthin supplemented groups than in the control group at 70 days.

**Fig 4:**
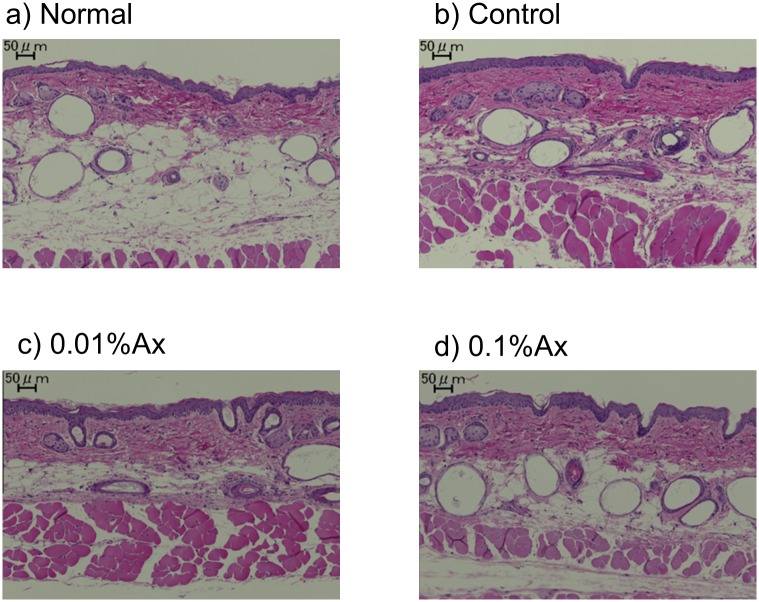
Photographs of sections of dorsal skin in hairless mice stained with hematoxylin and eosin.

Fig 4 shows the dorsal skin sections stained with H&E. The thickness of the epidermis is generally used as a parameter to reflect photoaging in skin. In this study, dietary astasanthin did not affect the increase of thickness of epidermis caused by UVA radiation.

### Accumulation of dietary astaxanthin in plasma and skin

**Fig 5:**
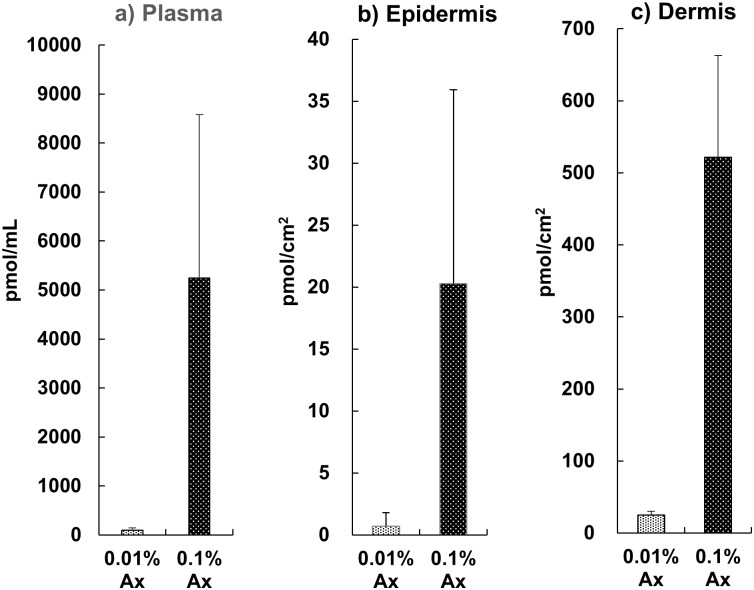
Astaxanthin concentration in the plasma and skin (epidermis and dermis) of the hairless mice. Data represent the means ± SD (n = 5). Bars with matching letters are significantly different from each other at P < 0.05.

Astaxanthin was detected in the plasma by using HPLC analysis after dietary supplementation for 70 days. The plasma concentration of astaxanthin in the 0.1% astaxanthin group was significantly higher than that in the 0.01% astaxanthin group. We confirmed that dietary astaxanthin was also able to reach the skin. As shown in Fig 5, astaxanthin was dose-dependently accumulated in the skin of mice, similar to the pattern observed in plasma. In addition, we found that the concentration of astaxanthin in the dermis was approximately 20–30 times higher than that in the epidermis per square millimeter of skin area.

### Effect of astaxanthin on mRNA expression in the skin

**Fig 6:**
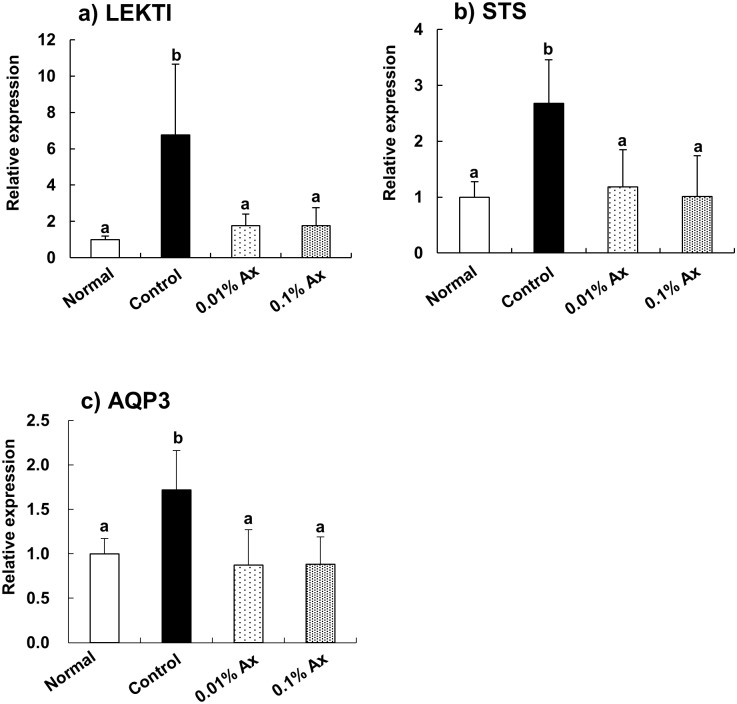
Effect of dietary astaxanthin on mRNA expression in the epidermis of hairless mice irradiated by UVA. Data represent the means ± SD (n = 5). Bars with matching letters are significantly different from each other at P < 0.05.

**Fig 7:**
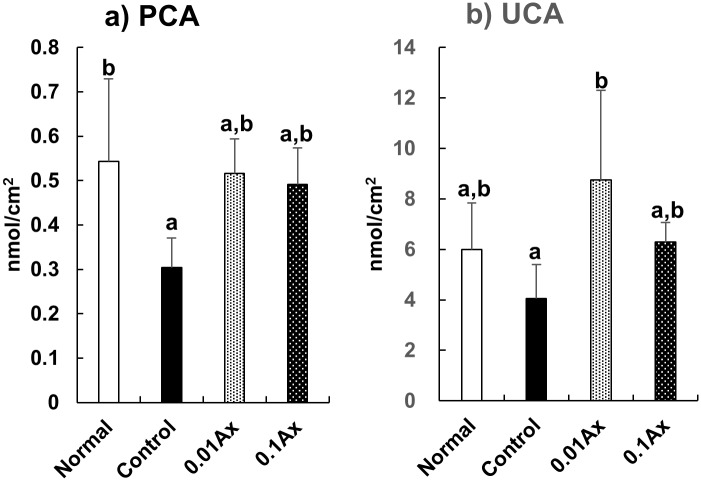
Effect of dietary astaxanthin on the NMF contents in the epidermis of hairless mice irradiated by UVA. Data represent the means ± SD (n = 5). Bars with matching letters are significantly different from each other at P < 0.05.

The effects of astaxanthin on gene expression related to barrier function in the epidermis were analyzed by using real-time RT-PCR. We found that the mRNA expression levels of lympho-epithelial Kazal-type-related inhibitor (LEKTI), steroid sulfatase (STS), and aquaporin 3 (AOP3) were significantly increased by UVA irradiation for 70 days. Dietary astaxanthin significantly suppressed these increases in mRNA expression to control levels (Fig 6). The serine protease inhibitor LEKTI, which is encoded by *SPINK5*, is expressed in the most differentiated viable layers of stratified epithelial tissue and inhibits serine proteases involving the human kallikrein (KLK)-related peptidases including KLK5, KLK5, and KLK14 [28]. These serine proteases are key proteases involved in desquamation and contribute to the production of natural moisturizing factors (NMFs) from pro-filaggrin [29]. Thus, we measured the contents of pyroglutamic acid (PCA) and urocanic acid (UCA), which are the major NMFs in the epidermis, by HPLC analysis. UVA exposure significantly decreased the content of PCA in the epidermis, and the content in the 0.01% and 0.1% astaxanthin groups demonstrated a trend toward improvement (Fig 7a). By contrast, the UCA content in the 0.01% astaxanthin group was significantly higher than that in the control group, while the UCA content in the normal and 0.1% astaxanthin groups was not significantly different from that in either the control or 0.01% astaxanthin groups (Fig 7b).

**Fig 8:**
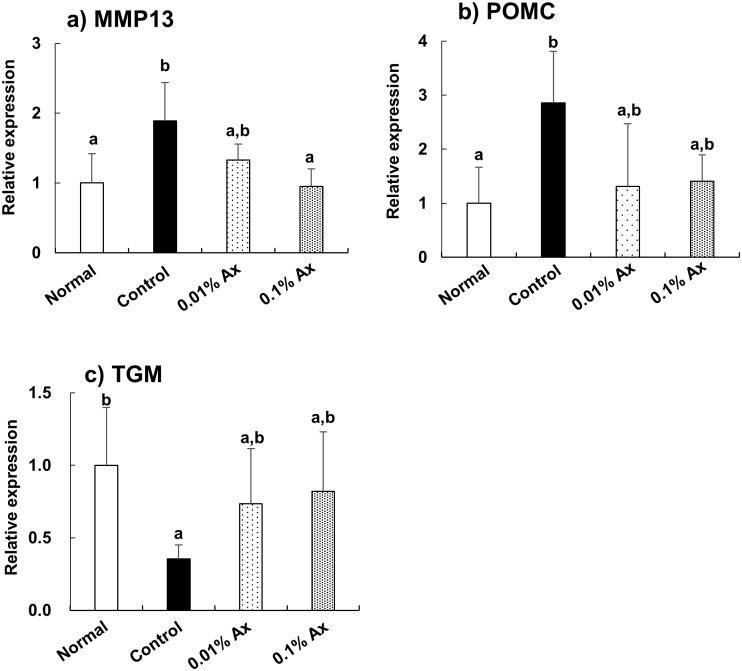
Effect of dietary astaxanthin on mRNA expression in the dermis of hairless mice irradiated by UVA. Data represent the means ± SD (n = 5). Bars with matching letters are significantly different from each other at P < 0.05.

In the dermis, the mRNA expression of matrix metalloprotease 13 (MMP13) was increased by UVA irradiation, and this increase was significantly suppressed in the 0.1% astaxanthin group (Fig 8a). The mRNA expression pattern of pro-opiomelanocortin (POMC) was similar to that of MMP13, but the effect of astaxanthin was not significant (Fig 8b). On the other hand, dietary astaxanthin tended to suppress the decrease of transglutaminase 2 (TGM2) mRNA expression by UVA irradiation (Fig 8c).

## Discussion

Diverse UV radiation plays an important role in the skin and regulates body homeostasis both on the local [30–32] and systemic levels [33–35]. To our knowledge, this is one of the first in vivo studies evaluating the protective effect of dietary astaxanthin against skin photoaging induced by UVA radiation. Intrinsic and chronological skin aging is characterized by atrophy of the skin with loss of elasticity and slowed metabolic activity. Quantitatively different from intrinsic aging, skin photoaging, which is clinically characterized by dryness, pigmentation, laxity, and deep wrinkling, occurs as a result of the accumulation of environmental damage, particularly exposure to ultraviolet radiation [3, 4]. It was suggested that exposure to UVA contributes to photodamage in human skin, because UVA accounts for approximately 95% of personal exposure to solar radiation [8]. In this study, we found that dietary supplementation with astaxanthin effectively prevented features of photoaging, such as the increase of TEWL and wrinkle formation, in the dorsal skin of mice exposed to UVA irradiation. Based on the body weight, the calculated astaxanthin intake in the condition of this study was 20–200 mg per day for human. However, it seems that lower dose is enough to be effective for human, because the absorption of carotenoids in mice is generally smaller than in human [36]. It has been reported that the concentration of plasma astaxanthin in human after 3 mg/day ingestion of astaxanthin for 12-week was 62±25 pmol/mL, as similar as the plasma astaxanthin level of 0.01% Ax group in this study (92±48 pmol/mL) [37].

Highly differentiated flattened keratinocytes, referred to as corneocytes, are the building blocks of the epidermal barrier. NMFs generated by the proteolysis of filaggrin are essential for the retention of water within corneocytes, and result in their optimal hydration and swelling [38]. Profilaggrin, the precursor of filaggrin, undergoes proteolysis by KLK5 to form filaggrin monomers [39], and filaggrin is degraded into small peptides and then into free amino acids through a multistep processes involving caspase-14 [40, 41]. KLK7 cleaves procaspase-14 to produce a secondary form that subsequently mediates conventional procaspase-14 activation [42]. LEKTI is a potent inhibitor of not only KLK5, KLK7, and KLK14 but also caspase-14 [43]. By contrast, the balance between the activity of proteases such as KLK7 and protease inhibitors such as LEKTI determine the rate of desquamation (corneocyte shedding) [28]. Therefore, epidermal features of photoaging might be partly caused by the UV-induced LEKTI expression. Our results indicated that dietary astaxanthin effectively suppressed the induction of LEKTI by chronic UVA irradiation. In addition, although there was individual variation, the decrease of NMF contents in the epidermis by UVA exposure appeared to be prevented by dietary astaxanthin.

In normal epidermis, cholesterol sulfate is generated by cholesterol sulfotransferase, but desulfated in the outer epidermis by STS, forming a ‘cholesterol sulfate cycle’ that potently regulates epidermal differentiation, barrier function, and desquamation [44]. It has been proposed that cholesterol is first sulfated in the lower epidermis, and then desulfated back to cholesterol in the outer epidermis [45] Disruption of this cholesterol sulfate cycle by chronic UVA irradiation might partly account for the abnormal desquamation of photoaging, and dietary astaxanthin may contribute to improving this disruption.

AQP3, which is a water/glycerol-transporting channel, is expressed in keratinocytes of the epidermis [46]. It has been suggested that AQP3 upregulation is involved in keratinocyte proliferation, epidermal hyperplasia, and barrier disruption in skin disorders [47]. In fact, strong expression of AQP3 was detected in both the stratum basale and the stratum spinosum in acute and chronic atopic eczema, although epidermal AQP3 was expressed weakly and mainly found in the stratum basale in the case of normal condition [48]. The increased expression and altered cellular distribution of AQP3 found in eczema may contribute to water loss. Therefore, the increase of AQP3 mRNA expression in the epidermis of hairless mice by UVA irradiation observed in this study might be involved in skin barrier disruption caused by photoaging.

UV radiation induces matrix metalloprotease 1 (MMP1) that leads to collagen damage, which is one of the hallmarks of photoaging [49]. However, rodents lack the MMP-1 gene, which appears to be functionally replaced by MMP13 [50]. The preventive effect of dietary astaxanthin on wrinkle formation could be due to the suppression of MMP induction by UVA irradiation. Our results are consistent with a previous report that UVA irradiation increased POMC expression in human keratinocytes [51] and decreased TGM2 expression in human skin fibroblasts [52]. In this study, dietary astaxanthin tended to suppress both the increase of PMOC and the decrease of TGM2 by UVA irradiation. The melanocyte-stimulating hormones (α-, β-, and γ-MSH), which are processed by distinct members of the prohormone convertase family, are derived from POMC [53, 54]. α-MSH suppresses the expression of collagen in human dermal fibroblasts [55]. TGM2 identified as a stable interaction partner of collagen VII is an important enzyme for protein cross-linking. The reduction of the activity of TGM2 accounts for the decrease of adhesion, the reduction of cross-linking of the extracellular matrix, and the decrease of epidermal–dermal integrity [56].

Most xanthophylls from dietary sources, including astaxanthin, are present in an esterified form. There are many reports suggesting that fatty acid esters of xanthophylls can be hydrolyzed in the digestive tract, because no ester form of dietary xanthophyll has been detected in blood after oral ingestion [57, 58]. Consistent with these previous findings, only the free form of astaxanthin was detected in the plasma and skin after supplementation with mono-esterified astaxanthin for 70 days in this study. Petri and Lundebye reported that the accumulation of dietary astaxanthin in hairless skin of the tail was much higher than those in other tissues. And color changes on the tail skin by the reflectance measurements using a portable spectrophotometer was highly correlated with the concentration of dietary astaxanthin [59]. In the present study, we confirmed that dietary astaxanthin reached not only the dermis but also the epidermis by using HPLC-PDA analysis. Our data supports the hypothesis that the accumulation of dietary astaxanthin in the skin could be sufficient to play a preventive role against UV damage.

Taken together, our study indicates the protective effects of dietary astaxanthin against features of photoaging induced by UVA radiation, such as impaired barrier function and wrinkling in the skin. We found that dietary astaxanthin accumulates in the skin and may prevent the effects of UVA irradiation on filaggrin metabolism and desquamation in the epidermis and the matrix in the dermis. Our results underscore the potential for astaxanthin to be further developed as a nutraceutical against photoaging.

## Supporting information

S1 Table Real time RT-PCR primers used for quantification od mouse mRNA. (XLSX)
